# Cardiopulmonary exercise test: A 20-year (2002-2021) bibliometric analysis

**DOI:** 10.3389/fcvm.2022.982351

**Published:** 2022-08-15

**Authors:** Lei Song, Hua Qu, Jinwen Luo, Wenting Wang, Liying Zheng, Mei Xue, Dazhuo Shi

**Affiliations:** ^1^Graduate School, Beijing University of Chinese Medicine, Beijing, China; ^2^Center of Cardiovascular Disease, Xiyuan Hospital, China Academy of Chinese Medical Sciences, Beijing, China; ^3^National Clinical Research Center for Chinese Medicine Cardiology, Xiyuan Hospital, China Academy of Chinese Medical Sciences, Beijing, China; ^4^Xiyuan Hospital, China Academy of Chinese Medical Sciences, Beijing, China; ^5^Graduate School, China Academy of Chinese Medical Sciences, Beijing, China

**Keywords:** cardiopulmonary exercise test, bibliometric analysis, citation analysis, cardiopulmonary fitness, cardiovascular disease

## Abstract

**Background:**

The clinical application value of cardiopulmonary exercise test (CPET) has increasingly attracted attention, and related research has been increasing yearly. However, there is no summary analysis of the existing CPET literature. This is the first bibliometric analysis of publications in the CPET.

**Methods:**

CPET-related articles published between 2002 and 2021 were retrieved from the Web of Science Core Collection database. The search was limited to Articles and Reviews in English. CiteSpace software was used to conduct collaborative network analysis of countries/regions, institutions, authors, the co-occurrence of subject categories and keywords, and co-citation analysis of authors, journals, and references.

**Results:**

A total of 4,426 publications were identified. During the study period, the number of published articles increased yearly. Developed countries from the Americas and Europe led the field. The University of Milan was the most prolific institution, with Ross Arena and Wasserman K being the most prolific and co-cited authors in the field, respectively. Cardiovascular System & Cardiology and Respiratory System were the main areas involved. Moreover, heart failure, oxygen uptake, and prognostic value were the central themes.

**Conclusions:**

CPET had attracted widespread attention, and the number of publications will increase substantially according to the current growth trends. In the future, CPET is expected to be further adopted in large-scale clinical studies as a means of assessing the functional ability of patients to verify the efficacy of related interventions. High-quality evidence-based medical CPET-related indicators is expected to be used in clinical diseases risk prediction.

## Introduction

Exercise testing have many modalities to be utilized in different clinical situations, the widely used ones include 6-minute walk test, exercise-induced bronchospasm test, stair climbing, shuttle test and cardiopulmonary exercise test (1). Cardiopulmonary exercise test (CPET) is an exercise test that objectively and quantitatively assesses cardiopulmonary fitness (CRF) by measuring breathing gas metabolic parameters simultaneously (e.g., oxygen uptake, carbon dioxide excretion) during exercise (2). At present, CPET is the internationally recognized gold standard for evaluating CRF (3), which is defined as the ability of the circulatory and respiratory systems to provide oxygen to skeletal muscle during continuous physical activity. It is a comprehensive reflection of the body's exercise capacity (4), assessed by the quantifiable index of maximum oxygen uptake (VO_2_max). Many studies have shown that CRF is a strong independent predictor of chronic diseases, such as cardiovascular disease (5) and cancer (6), and a modifiable risk factor which is influenced by lifestyle and physical activity. Relevant studies have shown that, compared with traditional clinical risk factors for cardiovascular disease, CRF can better predict the health status and possible adverse events (7, 8). Previously, CPET was mainly performed by cardiopulmonary physiologists for physiological scientific research, but in recent years, more studies have found that CRF-related indicators have an important predictive role in disease prognosis (9–12), which has led to the extensive use of CPET in clinical practice (13). In 2016, the American Heart Association proposed in a scientific statement that CRF should be categorized as a clinical vital sign and routinely assessed in clinical practice (14).

Bibliometrics is a scientific research method that quantitatively analyzes all knowledge carriers (journal articles, books, etc.) through mathematical or statistical methods (15), and it can be applied to any discipline to quantitatively analyze the historical development and evolution trends of a specific research field. With the development of information technology, a series of knowledge mapping software based on bibliometric research methods have emerged, which can present complex knowledge entities and their interrelationships in the form of visual graphics and facilitates understanding of the knowledge structure system of a research field. The medical field is a major field of bibliometric methods application, and bibliometric studies on cardiovascular (16), respiratory (17), dermatology (18), oncology (19), infectious diseases (20) have been recognized. The literature search reveals that the number of bibliometric-related documents shows a trend of increasing yearly.

As the use of CPET in clinical practice has become more widespread, more research based on CPET have been conducted. Although traditional literature reviews can summarize and analyze CPET, it is still mainly limited to a specific topic that the author is concerned about and cannot provide a comprehensive analysis of the CPET field which can help the other researchers quickly understand the key research team, research results and related progress. This problem can be effectively solved by introducing a bibliometric approach to compile and analyze the published documents about CPET from the past 20 years. We found that no bibliometric study on CPET has been conducted, which prompted us to conduct this study.

## Methods

### Data acquisition and search strategy

The Web of Science (WoS, Clarivate Analytics, Philadelphia, PA, USA) database collected more than 12,000 international academic journals and is one of the most comprehensive and authoritative database platforms for the assessment of global academic resources (21). In addition, it has the function of citation index search, which is a necessary condition for bibliometric co-citation analysis. Therefore, the WoS database was selected as the data source for our study.

All literature was retrieved and exported from the Web of Science Core Collection (WoSCC) database with the search formula TS (Topic) = (“cardiopulmonary exercise test^*^”). The indices we searched include Science Citation Index Expanded (SCI-EXPANDED), Social Sciences Citation Index (SSCI), Arts & Humanities Citation Index (AHCI), Conference Proceedings Citation Index–Science (CPCI-S), Conference Proceedings Citation Index–Social Science & Humanities (CPCI-SSH), Emerging Sources Citation Index (ESCI), Current Chemical Reactions (CCR-EXPANDED), Index Chemicus (IC). The preliminary search yielded 6,270 records, and a total of 4,426 records were obtained. The data analysis set through the results refinement function of WoSCC database, with the setting of language: English, document type: Articles, Review Articles, and the time span from January 1, 2002, to December 31, 2021. 4,426 records were selected with “Full record with cited references,” exported in “the plain text file format,” and renamed in the form of “download^*^.txt" to ensure that they can be read correctly by CiteSpace software (22).

### Data extraction

A total of 4,426 documents were imported into CiteSpace (5.8.R3) software to remove duplicates. The acquired documents were then manually reviewed by two independent researchers to ensure relevance to the CPET research topic. When there is a dispute between two researchers, they need to read the original article together to reach a final agreement.

### Data analysis

Microsoft Excel 2019 (Microsoft Corporation, Redmond, WA, USA) software was used for the graphs of the number of publications per year. The analysis of knowledge mapping visualization was performed by the CiteSpace software, which is one of the most popular software for bibliometric analysis. It was developed by Prof. Chaomei Chen based on the Java programming language and can carry out collaborative network analysis, co-occurrence analysis, and co-citation analysis to reveal the collaborative networks of countries, institutions and authors, the distribution of subject areas, high-frequency keywords and core literature in a particular research area in the form of visual graphs or tables.

## Results

### Annual quantitative distribution of publications

**Figure 1:**
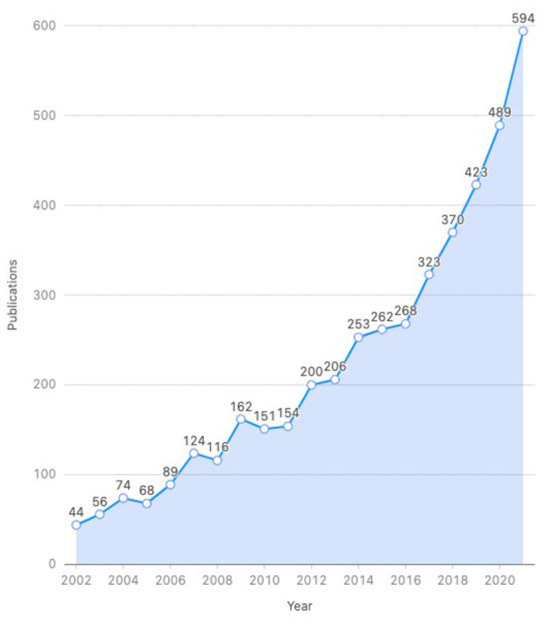
Annual number of publications about CPET.

The annual number of publications revealed the development trends of research in this field. As was shown in Figure 1, from 2002 to 2021, the number of publications related to CPET had been steadily increasing. A slow growth trend was observed from 2002 to 2017. In contrast, it had achieved a rapid increase from 2017 (323) to 2021 (594), which indicated that the CPET had received increasing attention in the last 5 years.

The 4,426 documents contain 4,180 articles and 246 reviews. Articles accounted for approximately 95% of the document type, indicating a greater focus on original research such as clinical trials or case reports.

### Collaborative network analysis

#### Country

Researchers from 104 countries published CPET-related articles, with the United States (1,135), England (513), Italy (475), Brazil (422), and Germany (339) being the top 5 countries (Table 1). It is worth noting that the total number of publications from the United States was more than twice that from England, which ranks second. At the same time, the United States had a high BC (betweenness centrality) value (BC = 0.62).

**Table 1:** Top 10 countries in terms of number of published CPET articles.

**Rank**	**Country**	**Count**	**BC**	**Year**
1	USA	1,135	0.62	2002
2	England	513	0.19	2007
3	Italy	475	0.00	2007
4	Brazil	422	0.00	2007
5	Germany	339	0.00	2007
6	Canada	253	0.88	2008
7	Japan	243	0.00	2007
8	Netherlands	197	0.24	2008
9	France	170	0.78	2008
10	Belgium	160	0.10	2007

*BC, betweenness centrality*.

**Figure 2:**
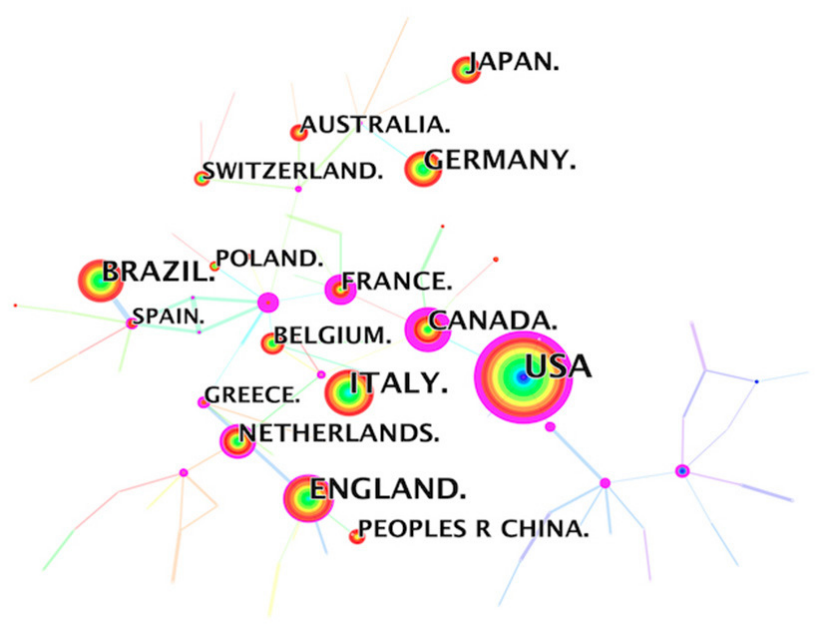
Country collaborative network analysis.

Figure 2 showed that each node represents a country, the node size is positively correlated with the frequency of co-occurrence in different country. The line between two nodes represents the co-occurrence relationship between two countries, and the thickness of the line indicates the strength of the relationship. The color of the rings and lines around the node indicate the year in which the country or relationship first appeared. The purple circles around some nodes indicate betweenness centrality (BC), which shows the importance of a node in the whole network. Nodes with a BC value ≥0.1 are marked in the network with a purple ring. The thickness of the purple ring is proportional to its BC value. The first year of appearance of the United States was 2002. It is 4–5 years earlier than the other countries in the top 10, indicating that the United States had taken the lead in CPET-related research on a global scale and the research interest was very high. The research results from the United States played an important role in the global collaborative research network. In addition, Canada (BC = 0.88) and France (BC = 0.78) also had a high BC value, indicating strong collaborative relationships with other countries. Italy, Brazil, Germany and Japan had low BC values, indicating that these countries have weaker cooperative relations with other countries, as shown in Figure 2 that they were located on the edge of the network structure. However, they were ranked in top 10 in terms of the number of publications, which reflects the great contributions these countries had made in this research field.

#### Institutions

CPET-related research involved 480 institutions worldwide, and six of the top 10 institutions in terms of number of articles published were from the United States, which once again highlight the strong influence of the United States in this field. The top three institutions were the University of Milan (191, Italy), Stanford University (114, USA), and the University of São Paulo (105, Brazil) (Table 2).

**Table 2:** Top 10 institutions based on the number of CPET publications.

**Rank**	**Institution**	**Count**	**BC**	**Country**	**Year**
1	University of Milan	191	0.10	Italy	2002
2	Stanford University	114	0.05	USA	2004
3	University of São Paulo	105	0.00	Brazil	2002
4	Mayo Clinic	90	0.02	USA	2002
5	University of Illinois	90	0.05	USA	2013
6	Istituto di Ricovero e Cura a Carattere Scientifico	85	0.22	Italy	2002
7	Brigham and Women's Hospital	73	0.27	USA	2005
8	Technical University of Munich	70	0.01	Germany	2005
9	Virginia Commonwealth University	70	0.19	USA	2004
10	Duke University	68	0.28	USA	2002

*BC, betweenness centrality*.

**Figure 3:**
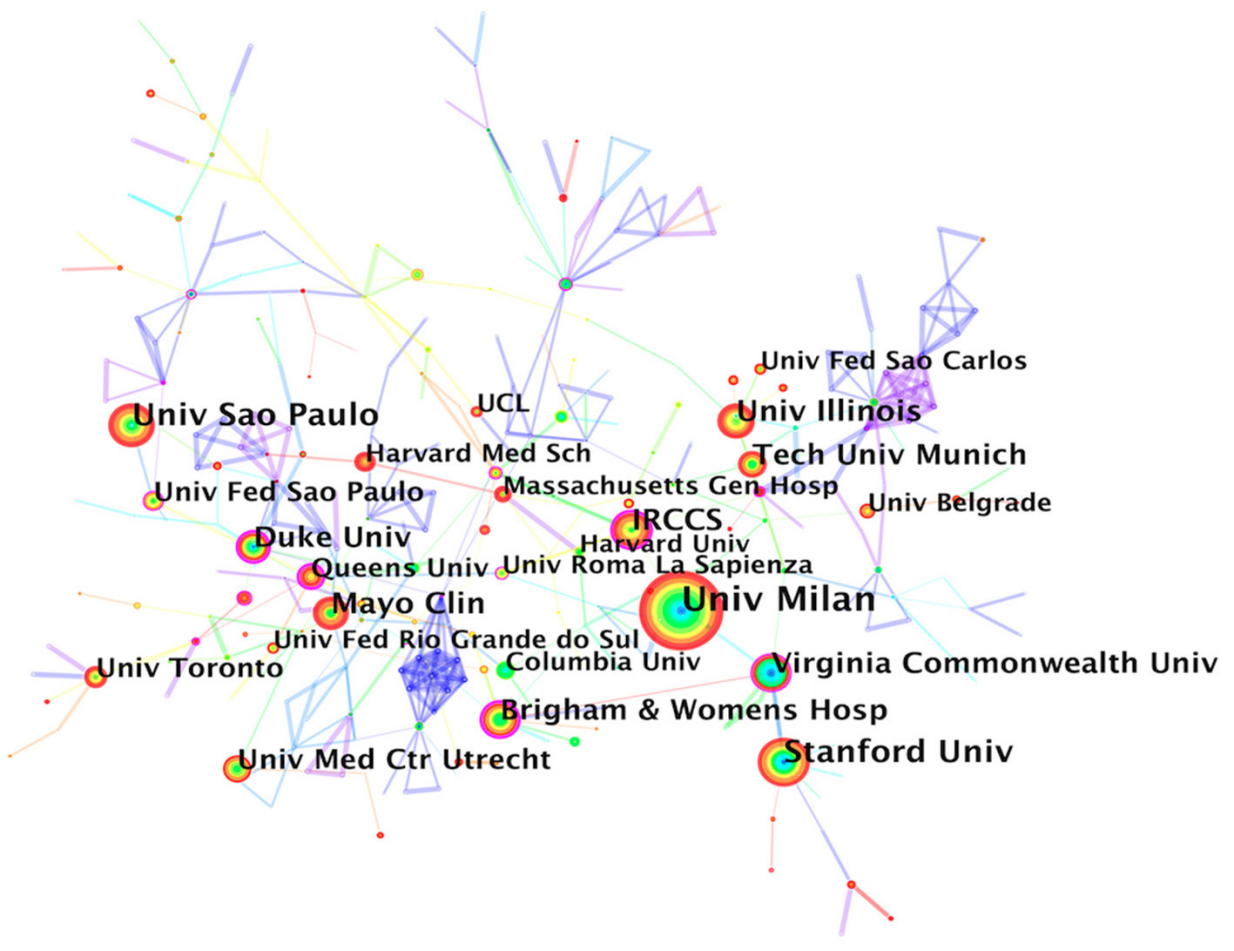
Institution collaborative network analysis.

The 1st ranked University of Milan had almost 80 more articles than the 2nd ranked Stanford University, indicating a huge lead. Interestingly, none of the top five institutions had a BC value >0.1, indicating that the cooperation between the top research institutions was not close, and they mainly conducted CPET-related research independently (Figure 3).

#### Authors

**Figure 4:**
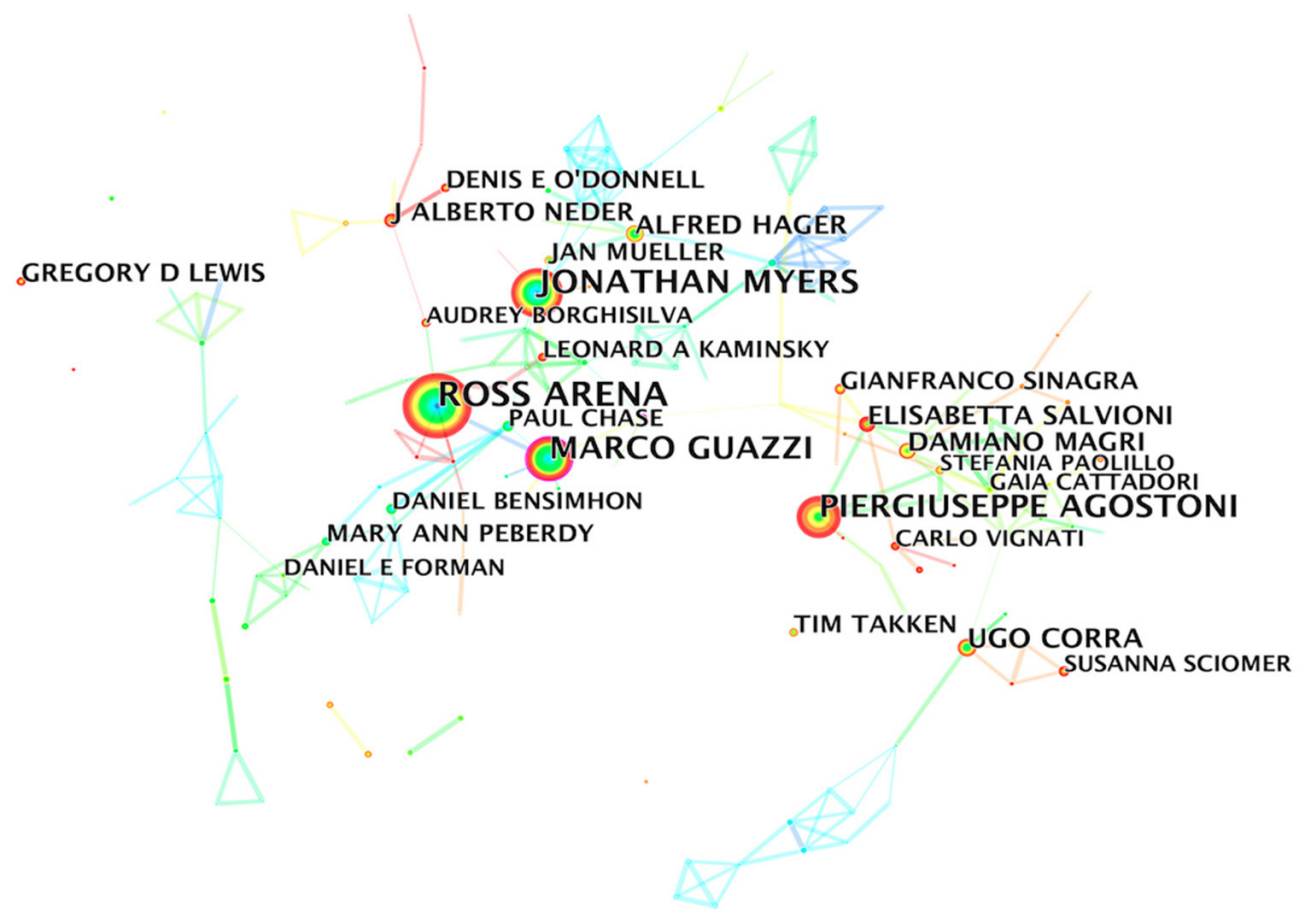
Author collaborative network analysis.

The analysis of the author collaborative network showed that Ross Arena (142) had the most published articles, followed by Jonathan Myers (110), Marco Guazzi (96), and Piergiuseppe Agostoni (92) (Table 3), and the number of articles per capita had reached over 90, which was the highest ranking. The 5th to 10th authors had more than twice the number of published papers per capita and constitute the most representative high-yield author team in the CPET field. Further, the BC values of the top 10 authors were not high, which once again confirmed that the research cooperation relationship related to CPET was not strong. In Figure 4, each node represent an author. Nodes with usage bursts are visualized by red rings, indicating that the author appeared at a high frequency during a certain period. Most of the top 10 authors had high burst values, suggesting that their research directions were well correlated with CPET topics.

**Table 3:** Top 10 authors based on the number of CPET publications.

**Rank**	**Author**	**Count**	**Burst**	**BC**	**Year**
1	Ross Arena	142	–	0.03	2006
2	Jonathan Myers	110	3.91	0.04	2006
3	Marco Guazzi	96	3.18	0.10	2006
4	Piergiuseppe Agostoni	92	8.91	0.01	2008
5	Ugo Corra	45	–	0.03	2006
6	Alfred Hager	41	4.33	0.00	2008
7	Damiano Magri	39	4.88	0.05	2008
8	Elisabetta Salvioni	39	6.21	0.07	2012
9	J Alberto Neder	37	12.01	0.02	2016
10	Gregory D Lewis	36	5.83	0.00	2010

*BC, betweenness centrality*.

### Co-occurrence analysis

#### Subject categories

Supplementary Table 1 showed that Cardiovascular System & Cardiology (1948) was a key discipline in the CPET field, and the number of publications under this discipline category far exceeded that of other disciplines. Respiratory System (776), and General & Internal Medicine (404) were ranked 2nd and 3rd respectively. In addition, Sport Sciences, Physiology, Surgery, Pediatrics, Rehabilitation, Research & Experimental Medicine, and Critical Care Medicine were also applied subjects of CPET in clinical practice.

It is worth pointing out that Critical Care Medicine had a significantly high BC value (BC=0.92), suggesting that it was the connection between other sub-subject categories (Supplementary Figure 1).

#### Keywords

Keywords are the highly refined content of the full text, which reflects the article topic. Table 4 showed the top 20 keywords with co-occurrence frequency in CPET field. Among them, heart failure, exercise capacity, quality of life, oxygen uptake all co-occurred more than 300 times, and the BC values of keywords such as morality, physical activity, survival, risk, oxygen consumption, adult, anaerobic threshold, and gas exchange were all ≥0.1, indicating that they played an important role in connecting other keywords in the whole keyword network.

**Table 4:** Top 20 keywords on CPET.

**Rank**	**Keywords**	**Count**	**BC**	**Rank**	**Keywords**	**Count**	**BC**
1	Heart failure	805	0.07	11	performance	323	0.03
2	Capacity	644	0.09	12	association	313	0.02
3	Exercise	623	0.03	13	survival	287	0.38
4	Mortality	566	0.15	14	risk	271	0.11
5	Cardiopulmonary exercise testing	554	0.08	15	oxygen consumption	268	0.18
6	Exercise capacity	474	0.03	16	adult	268	0.24
7	Disease	389	0.03	17	anaerobic threshold	262	0.31
8	Physical activity	369	0.14	18	gas exchange	250	0.29
9	Quality of life	358	0.04	19	recommendation	242	0.00
10	Oxygen uptake	328	0.10	20	children	242	0.03

*BC, betweenness centrality*.

**Figure 5:**
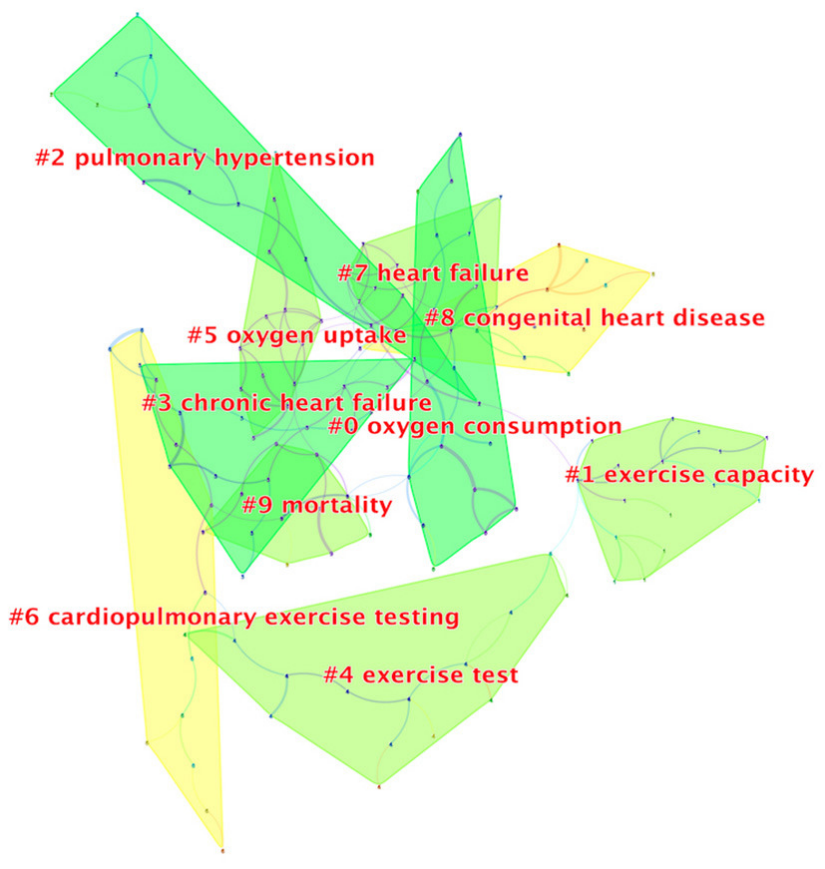
Keyword cluster analysis.

To further determine the relationships among keywords, we used the log-likelihood rate (LLR) algorithm model to perform a cluster analysis and extracted the top 10 cluster labels based on the keyword fields. Modularity Q=0.7894, Silhouettes S=0.9455, indicating that the structure of the clusters was significant, and the clustering relationship was highly credible. Smaller values of the cluster labels indicate a larger number of keywords within the cluster. The top 10 clustering labels were: oxygen consumption (#0), exercise capacity (#1), pulmonary hypertension (#2), chronic heart failure (#3), exercise test (#4), oxygen uptake (#5), cardiopulmonary exercise testing (#6), heart failure (#7), congenital heart disease (#8), and mortality (#9). The cluster labels generally reflected three major aspects: the clinical significance of CPET, the evaluation indicators and the diseases in which it is mainly applied in clinical practice (Figure 5).

**Figure 6:**
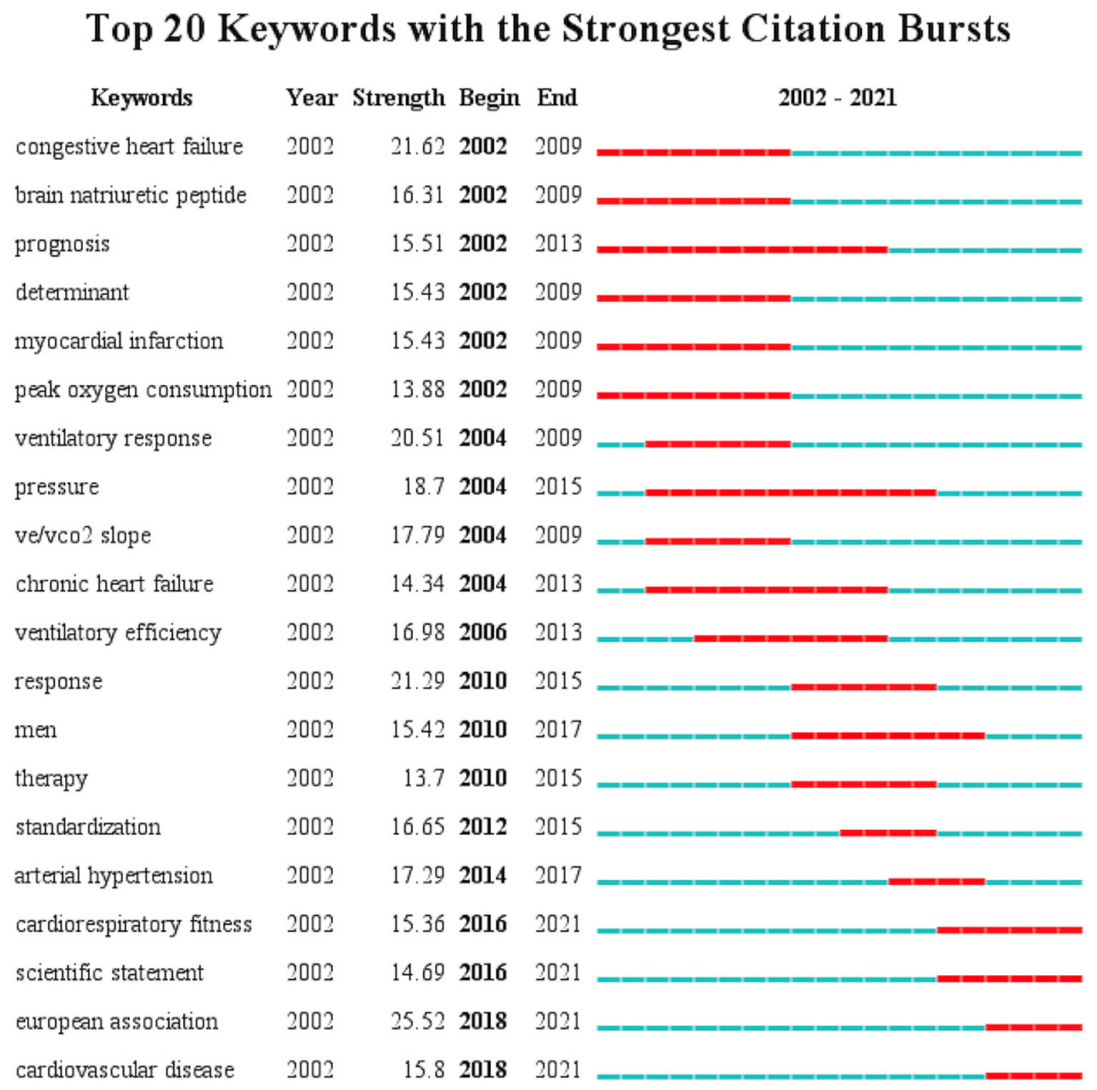
Top 20 keywords in burst analysis.

Keywords burst analysis refers to a surge in the frequency of a keyword in a certain period, indicating that the keyword had received high attention from researchers in that period. The burst strength represents the intensity of the rapid growth of a keyword. Based on the keywords burst analysis, research hotspots in different periods during the development of CPET can be discovered. As shown in Figure 6, between 2002 and 2009, congenital heart disease, BNP and heart failure, and myocardial infarction were the main research hotspots, and peak oxygen uptake was the main evaluation indication of CPET. Between 2004 and 2014, many studies focused on the evaluation of other indications provided by CPET, such as the index of ventilation efficiency over the min ventilation-carbon dioxide production (VE/VCO_2_) slope. In the last 5 years, the concept of CRF had been introduced and attracted continuous and widespread attention, CPET had been widely recommended by clinical guidelines for cardiovascular diseases. Cardiovascular diseases were still the main application area of CPET and this continues to be a main research interest.

### Co-citation analysis

#### Author

**Figure 7:**
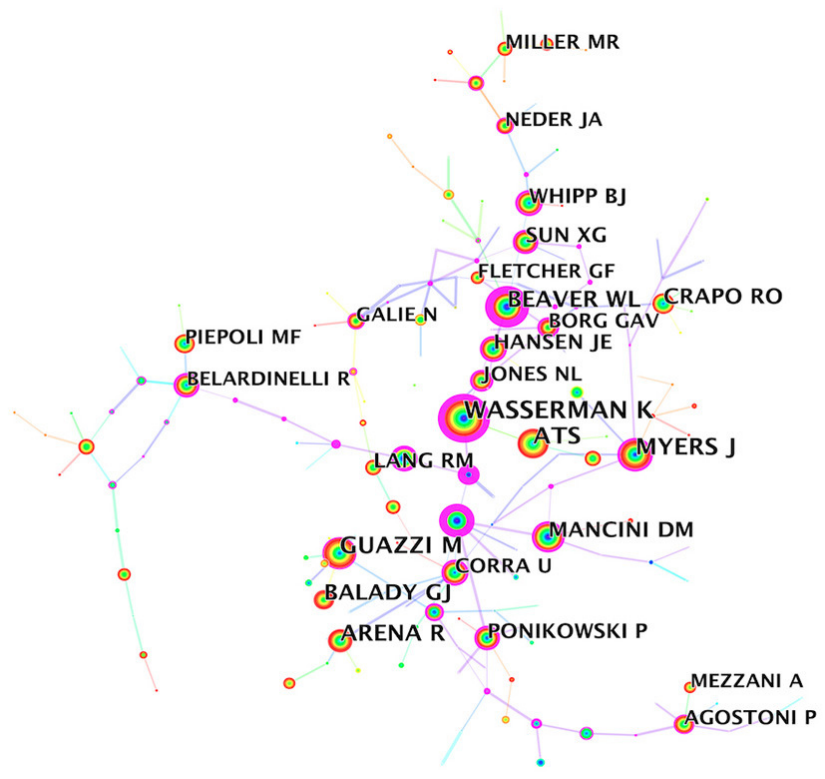
Author co-cited analysis.

When two authors' papers are simultaneously cited by a third author's papers, it indicates that there is a co-citation relationship between these two authors. Table 5 showed the top 10 authors by co-citation frequency. Wasseman K had an absolute lead in co-citation frequency (1,078) and a high BC value (0.91), indicating that his research had a great influence in the CPET field. The second was the American Thoracic Society rather than an independent author because CiteSpace software only extracts the first author by default when conducting author co-citation analysis. The high citation frequency was mainly related to the clinical guidelines it published as the author. It is worth pointing out that the authors ranked 3rd to 5th: Guazzi M, Myers J, Arena R were also the top three authors in the author co-occurrence analysis, indicating that they had a steady contribution to the CPET field. It should also be noted that the 6th ranked author Beaver WL had a high BC value (1.00), suggesting that his research was widely cited throughout the co-citation network (Figure 7).

**Table 5:** Top 10 authors ranked by co-cited frequency on CPET publications.

**Rank**	**Co-cited author**	**Co-cited frequency**	**BC**
1	Wasserman K	1,078	0.91
2	American Thoracic Society	789	0.04
3	Guazzi M	716	0.13
4	Myers J	669	0.28
5	Arena R	637	0.09
6	Beaver WL	595	1.00
7	Balady GJ	490	0.00
8	Mancini DM	415	0.39
9	Crapo RO	385	0.07
10	Whipp BJ	365	0.26

*BC, betweenness centrality*.

#### Journal

It can be seen from Table 6 that 50% of the top 10 journals ranked by co-cited frequency were professional journals in the cardiovascular field, and the rest of the journals categories included general internal medicine, physiology, sport sciences, and critical care medicine. Half of the journals had an impact factor of ≥10, and 90% are JCR Q1 or Q2.

**Table 6:** Top 10 journals ranked by co-cited frequency on CPET publications.

**Rank**	**Journal**	**Co-cited frequency**	**BC**	**Impact factor (2020)**	**Category**	**Quartile in category (JCR)**
1	Circulation	3,191	1.15	29.690	Cardiac and Cardiovascular Systems	Q1
2	Journal of the American College of Cardiology	2,617	0.81	24.093	Cardiac and Cardiovascular Systems	Q1
3	Chest	2,057	0.71	9.410	Critical Care Medicine	Q1
4	European Heart Journal	2,035	0.36	29.983	Cardiac and Cardiovascular Systems	Q1
5	American Journal of Cardiology	1,920	0.32	2.778	Cardiac and Cardiovascular Systems	Q3
6	Journal of Applied Physiology	1,871	0.31	3.532	Physiology	Q2
7	American Journal of Respiratory and Critical Care Medicine	1,801	0.46	21.405	Critical Care Medicine	Q1
8	The New England Journal of Medicine	1664	0.11	91.253	Medicine, General and Internal	Q1
9	Medicine and Science in Sports and Exercise	1,622	0.18	5.411	Sport Sciences	Q1
10	American Heart Journal	1,521	0.18	4.749	Cardiac and Cardiovascular Systems	Q2

*BC, betweenness centrality*.

In addition, the BC value of 10 journals were all ≥0.1 (Supplementary Figure 2). All these findings indicated that the clinical application of CPET was a prominent research topic, and the CPET-related papers published in these high-level journals also provided sufficient theoretical and data for the clinical application of CPET.

#### References

As was shown in Table 7, the top 10 most co-cited documents were clinical guidelines, scientific statements, or reviews. These documents were published after 2010, indicating that CPET had been gradually included in clinical guidelines and recommended for use in related diseases since 2010. It also reflected that researchers had gradually recognized the value of CPET in clinical auxiliary diagnosis and risk assessment. At the same time, relevant societies had formulated standard operating procedures for CPET in adults and introduced standardized guidance documents for the interpretation of CPET indications in specific populations, to further promote CPET's clinical application.

**Table 7:** Top 10 references ranked by co-cited frequency on CPET.

**Rank**	**Title**	**Reference type**	**Co-cited frequency**	**Year**	**BC**	**Impact factor (2020)**	**Quartile in category (JCR)**
1	2016 ESC Guidelines for the diagnosis and treatment of acute and chronic heart failure: The Task Force for the diagnosis and treatment of acute and chronic heart failure of the European Society of Cardiology (ESC)Developed with the special contribution of the Heart Failure Association (HFA) of the ESC (23)	Practice Guideline	143	2016	0.08	29.983	Q1
2	Clinician's Guide to cardiopulmonary exercise testing in adults: a scientific statement from the American Heart Association (24)	Review	124	2010	0.23	29.690	Q1
3	2016 Focused Update: Clinical Recommendations for Cardiopulmonary Exercise Testing Data Assessment in Specific Patient Populations (25)	Scientific Statements	113	2016	0.01	29.983	Q1
4	Importance of Assessing Cardiorespiratory Fitness in Clinical Practice: A Case for Fitness as a Clinical Vital Sign: A Scientific Statement From the American Heart Association (14)	Review	102	2016	0.10	29.690	Q1
5	2015 ESC/ERS Guidelines for the diagnosis and treatment of pulmonary hypertension: The Joint Task Force for the Diagnosis and Treatment of Pulmonary Hypertension of the European Society of Cardiology (ESC) and the European Respiratory Society (ERS): Endorsed by: Association for European Pediatric and Congenital Cardiology (AEPC), International Society for Heart and Lung Transplantation (ISHLT) (26)	Practice Guideline	96	2016	0.03	29.983	Q1
6	Recommendations for cardiac chamber quantification by echocardiography in adults: an update from the American Society of Echocardiography and the European Association of Cardiovascular Imaging (27)	Review	74	2015	0.00	5.251	Q2
7	EACPR/AHA Scientific Statement. Clinical recommendations for cardiopulmonary exercise testing data assessment in specific patient populations (28)	Practice Guideline	69	2012	0.03	29.690	Q1
8	Cardiopulmonary Exercise Testing: What Is its Value (29)	Review	69	2017	0.00	24.093	Q1
9	Cardiopulmonary Exercise Testing in Heart Failure (30)	Review	64	2016	0.19	12.035	Q1
10	ATS/ACCP Statement on cardiopulmonary exercise testing (31)	Review	61	2003	0.02	21.405	Q1

*BC, betweenness centrality*.

## Discussion

The number of articles published each year represents the level of activity in the development of a particular research field. The significant surge in the number of publications on the topic of CPET since 2017 indicated growing interest in the field. Clinicians gradually realized that CPET can provide important clinical auxiliary diagnostic information (29), so they were becoming more willing to perform CPET as part of a routine clinical examination. In addition, it may also because that CPET is gradually moving worldwide, and all regions of the world are vigorously promoting CPET-related research.

The country collaboration network showed that countries with strong comprehensive economic strength in North America and Europe dominated the CPET research field, followed by China, Japan, and Australia, but African countries had fewer publications, revealing the current uneven global development trends in the CPET field. This may be related to the higher national health awareness in developed countries such as American, England, and Italy. It is worth noting that the CPET instruments commonly used in clinical practice are also mainly manufactured by European and North American countries.

Almost all top 10 research institutions in terms of collaborative publications were the top 10 universities or their affiliated medical institutions in Europe countries and the United States, which indicated that top research and medical talents from these countries were driving the development in the CPET field. However, there were no institutions from Asia, Australia, or Africa among the top 10 institutions, indicating that the institutional partnership between Europe and the United States and other regional countries was not strong, which may be due to language factors or different health awareness among people in different countries. Tongji University from China ranked 30th, with a frequency of 31. In recent years, its number of publications had increased rapidly, showing a great development trend, which is expected to become a bridge for cross-regional cooperation with institutions of European and American countries.

Author collaboration network analysis suggested a non-tight collaboration among the core authors. Jonathan Myers, Marco Guazzi, and Ross Arena were the core researchers in the CPET field because of their high number and citations of published papers. However, it should also be noted that although Wasserman K did not have a huge number of publications, they ranked first in number of citations. He was an American physiologist, the founder of the Wasserman 9-Panel Plot (32) (the current standard layout for the presentation of CPET gas metabolism data), and one of the first people to propose the use of exercise tests to detect the coordination of cardiorespiratory metabolism (33, 34). He was also the first person to propose the determination of anaerobic thresholds to measure health risk by detecting changes in the composition of exhaled gas during exercise (35, 36).

Subject co-occurrence analysis showed that cardiovascular, respiratory, and other traditional medical system diseases were the main applied subjects of CPET. Sport sciences and physiology were interdisciplinary subjects involved in CPET, because the operation of CPET (treadmill or ergometry bicycle) and the interpretation of the results require the participation of sports scientists and cardiopulmonary physiologists. They can evaluate patients' exercise ability according to CPET results and formulate corresponding exercise programs to guide patients' rehabilitation outside the hospital (37–39).

Keywords co-occurrence and cluster analysis revealed the research status and hotspots of CPET. Heart failure was a major application area of CPET (40–42). CPET was always used to determine the maximum heart rate during exercise to guide the patient's daily physical activity and to assess the impact of drugs or interventions on the patient's exercise capacity (43, 44). Indicators from CPET had an independent predictive effect on prognosis in patients with heart failure, whether used alone or in combination with other non-CPET indicators. However, the establishment of CPET-related prognostic indicators had undergone many years of development and optimization. The early proposed VO_2_ peak had been widely recognized for its predictive role in prognosis and was the gold standard for evaluating functional ability (45–47). Subsequently, to exclude the effects of obesity and β-blockers on VO_2_, the VE/VCO_2_ slope was also proved to have a good predictive effect (48–50). Furthermore, to exclude the influence of subjective motility of patients, related studies had proposed using log OUES (oxygen uptake efficiency slope) as the predictor of mortality (51–54), and some studies had proposed that excessive ventilation assessed over the first 3 min should be taken for prognostic stratification to patients unable to perform maximal CPET (55). What's more, peak circulatory power, the combined application of AT and VE/VCO_2_ slope, VO_2_ peak and VE/VCO_2_ had proven to be strong predictors of heart failure mortality risk (56, 57). The combination application of %VO_2_ and BNP can further optimize the risk stratification in patients with chronic heart failure (58, 59). In addition to heart failure, the routine application of CPET in clinical practice had expanded to other patient populations, including those with coronary artery disease (60), congenital heart disease (61), and pulmonary hypertension (62, 63).

Co-citation analysis of references showed three publications with BC>0.1, revealing three important turning points in CPET development. The first turning point occurred in 2010 (24) when the American Heart Association issued a scientific statement indicating that CPET can provide more clinical information than other standard exercise tests, especially in diagnosingcardiopulmonary system diseases. More importantly, the document clearly specified the calibration of instruments and analysis systems, the selection principles of different CPET protocols, quality control and supervision during the test and results interpretation in clinical practice. Thus, it provided direct and detailed guidance to clinicians, pioneered a standardized test protocol for CPET, and laid a good foundation for its wider application in different patient populations. The remaining two turning points both occurred in 2016. The first reviewed the predictive role of CPET indicators on heart failure prognosis, how to use CPET indicators for the risk stratification management of heart failure, and summarized the available studies related to CPET in heart failure (30). The second was a scientific statement from the American Heart Association that analyzed a series of research findings showing the association between CRF and health outcomes and recommends CRF as a predictor of health status (14). It also highlighted the importance of CRF in predicting cardiovascular mortality and its role in pre-surgical risk assessment and risk stratification for heart failure and stroke, and it is now widely used in the construction of disease risk prediction models. In addition to cardiovascular diseases, CPET also played an important role in metabolic diseases such as diabetes and cancer. In conclusion, these three key references can be seen as landmark events in CPET field, which had a profound impact on promoting the application of CPET in cardiovascular and other diseases.

It is worth noting that the top 10 references in the co-citation analysis were all clinical guidelines or reviews, indicating that highly co-cited original articles about CPET were still relatively lacking. High-quality clinical research using CPET as the main efficacy evaluation index in cardiovascular system disease is expected to become a promising direction for future development. In addition, how to further popularize the theoretical basis of CPET among clinicians and clarify its clinical benefits should also be studied.

## Limitation

Although this is the first bibliometric analysis on a CPET topic, there are some potential limitations in this study. Like other bibliometric analyses, the search results obtained in this study were limited by the selected database. To meet the format requirements of CiteSpace software for analysis, only the WoSCC database was used in this study, so some literature related to this topic may not be included. The timespan of publication for the included literature is: 2002 to 2021, which will also result in earlier papers not being included in the analysis, we limit the language to English, which may lead to these literatures published in other languages being ignored. With the publication of relevant scientific research results, the results of bibliometric analysis in this topic may change in the future, but a summary analysis of 20 years of related results in this field is also helpful for later researchers to understand the development overview of this field in a specific period.

## Conclusion

To the best of our knowledge, this is the first bibliometric study to summarize the results of the last 20 years of research on CPET in the WoSCC database, using the CiteSpace software. The results of this study can help new researchers to understand the major research institutions, famous researchers, classic articles, and development trends in this field. The CPET field is in a period of vigorous development, and relevant research results are emerging. The annual number of publications is expected to further increase. Ross Arena had published the most literature in this field, and Wasserman K was the author with the highest co-citation frequency, indicating that his research results had greatly contributed to the development of this field. The literature published in top international journals in the cardiovascular field had made an important contribution to advancing the development of CPET field, indicating that CPET had attracted widespread attention from the academic community. At present, the main clinical application of CPET is cardiovascular diseases, mainly heart failure, and its main roles include objective and quantitative assessment of patients' CRF and guidance of patients' functional exercise. High-quality clinical studies with large samples using cardiopulmonary exercise testing as the main efficacy evaluation index are promising directions for future research.

## Data availability statement

The original contributions presented in the study are included in the article/Supplementary material, further inquiries can be directed to the corresponding authors.

## Author contributions

DS and MX conceived the study and reviewed and revised the manuscript. LS collected the data and wrote the manuscript. HQ and JL re-examined the data. LS, WW, and LZ analyzed the data. All authors contributed to the article and approved the submitted version.

## Funding

This study was supported by National Key Research and Development Program of China (No. 2019YFC0840608), Major research project of scientific and technological innovation project of Chinese Academy of Chinese Medicine Sciences (No. CI2021A00913), and Innovation Team and Talents Cultivation Program of National Administration of Traditional Chinese Medicine (No. ZYYCXTD-C-202007).

## Conflict of interest

The authors declare that the research was conducted in the absence of any commercial or financial relationships that could be construed as a potential conflict of interest.

## Publisher's note

All claims expressed in this article are solely those of the authors and do not necessarily represent those of their affiliated organizations, or those of the publisher, the editors and the reviewers. Any product that may be evaluated in this article, or claim that may be made by its manufacturer, is not guaranteed or endorsed by the publisher.

## Supplementary material

Supplementary Figure 1 Subject categories co-occurrence analysis network.

Supplementary Figure 2 Journal co-cited analysis.
