# Supplementary material for: Cardiopulmonary exercise test: A 20-year (2002-2021) bibliometric analysis
Source: Front Cardiovasc Med. 2022 Aug 15;9:982351. doi: 10.3389/fcvm.2022.982351 (PMC9420934; doi:10.3389/fcvm.2022.982351)
Supplement: Supplementary file 1 [file Table_1.DOCX]

Supplementary Table 1. Top 10 subject categories related with CPET publications

| Rank | Subject category | Count | BC | Year |
| --- | --- | --- | --- | --- |
| 1 | Cardiovascular System & Cardiology | 1948 | 0.14 | 2002 |
| 2 | Respiratory System | 776 | 0.07 | 2002 |
| 3 | General & Internal Medicine | 404 | 0.33 | 2002 |
| 4 | Sport Sciences | 365 | 0.15 | 2002 |
| 5 | Physiology | 287 | 0.20 | 2002 |
| 6 | Surgery | 229 | 0.07 | 2002 |
| 7 | Pediatrics | 195 | 0.00 | 2002 |
| 8 | Rehabilitation | 167 | 0.03 | 2004 |
| 9 | Research & Experimental Medicine | 134 | 0.92 | 2002 |
| 10 | Critical Care Medicine | 129 | 0.10 | 2002 |

BC: betweenness centrality
