# Supplementary material for: Cardiopulmonary exercise test: A 20-year (2002-2021) bibliometric analysis
Source: Front Cardiovasc Med. 2022 Aug 15;9:982351. doi: 10.3389/fcvm.2022.982351 (PMC9420934; doi:10.3389/fcvm.2022.982351)
Supplement: Supplementary file 2 [file Data_Sheet_1.pdf]

References of historical work about Exercise Testing

Smith, E. F. (1897). The Lavoisier Monument. Science, 5(114), 403-404.

Stokes, M. A. (1991). Antoine Lavoisier and the study of respiration: 200 years old. Australian and New Zealand Journal of Surgery, 61(3), 229-232.

Karamanou, M., & Androutsos, G. (2013). Antoine-Laurent de Lavoisier (1743–1794) and the birth of respiratory physiology. Thorax, 68(10), 978-979.

Forbes, R. M. (1955). Nathan Zuntz: (October 7, 1847–March 23, 1920). The Journal of Nutrition, 57(1), 1-15.

Gunga, H. C., & Kirsch, K. A. (1995). Nathan Zuntz (1847-1920)--a German pioneer in high altitude physiology and aviation medicine, Part I: Biography. Aviation, space, and environmental medicine, 66(2), 168-171.

Gunga, H. C., & Kirsch, K. A. (1995). Nathan Zuntz (1847-1920)--a German pioneer in high altitude physiology and aviation medicine, Part II: Scientific work. Aviation, space, and environmental medicine, 66(2), 172–176.

Macfarlane, D. J. (2017). Open-circuit respirometry: a historical review of portable gas analysis systems. European journal of applied physiology, 117(12), 2369-2386.

Bolt, W., Knipping, H. W., Valentin, H., & Venrath, H. (1953). Indikationen zu chirurgischen Eingriffen am Herzen1. DMW-Deutsche Medizinische Wochenschrift, 78(15), 523-527.

Knipping, H. W., & Valentin, H. (1964). “Vita-Maxima” Problems in the Management of Cardiac Patients. Postgraduate Medicine, 35(1), 68-82.

Knipping, H. W. (1965). Kinesitherapy and sports in old age. Munchener Medizinische Wochenschrift (1950), 107(27), 1329-1334.

Astrand, I., & Lundman, T. (1968). The Exercise Electrocardiogram in Coronary Heart Disease: Its Prognostic Value. Scandinavian Journal of Clinical and Laboratory Investigation, 22(4), 301-306.

Astrand, I. (1969). Prognostic value of exercise electrocardiogram in older men. Scandinavian Journal of Clinical and Laboratory Investigation, 23(3), 271-276.

Astrand, P. O. (1973). Physiology of exercise and physical conditioning in normals. Schweizerische Medizinische Wochenschrift, 103(2), 41-45.

Astrand P. O. (1976). Physiological evaluation of an exercise test. Bibliotheca cardiologica, (36), 3–6.

Åstrand, P. O. (1976). Quantification of exercise capability and evaluation of physical capacity in man. Progress in cardiovascular diseases, 19(1), 51-67.
