# Supplementary figures and images for: Cardiopulmonary exercise test: A 20-year (2002-2021) bibliometric analysis
Source: Front Cardiovasc Med. 2022 Aug 15;9:982351. doi: 10.3389/fcvm.2022.982351 (PMC9420934; doi:10.3389/fcvm.2022.982351)

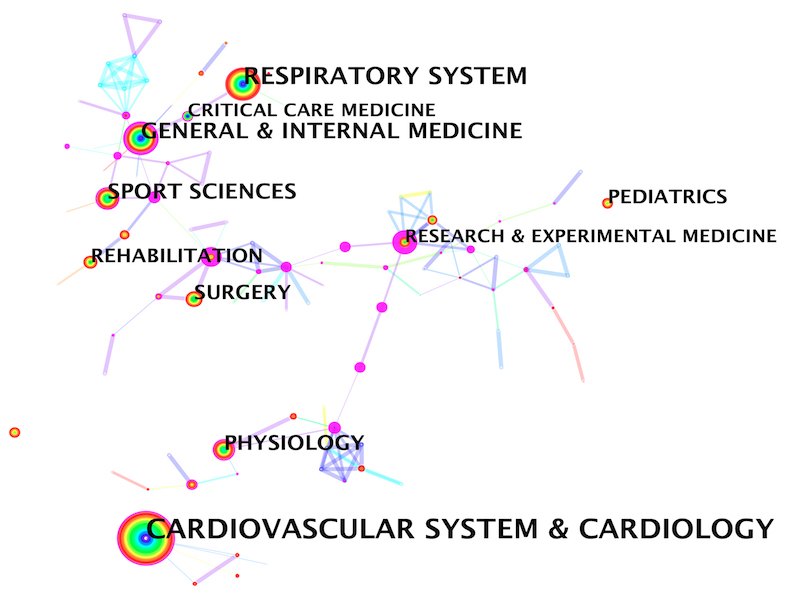

Supplement: Supplementary Figure 1 — Subject categories co-occurrence analysis network. [file Image_1.JPEG]

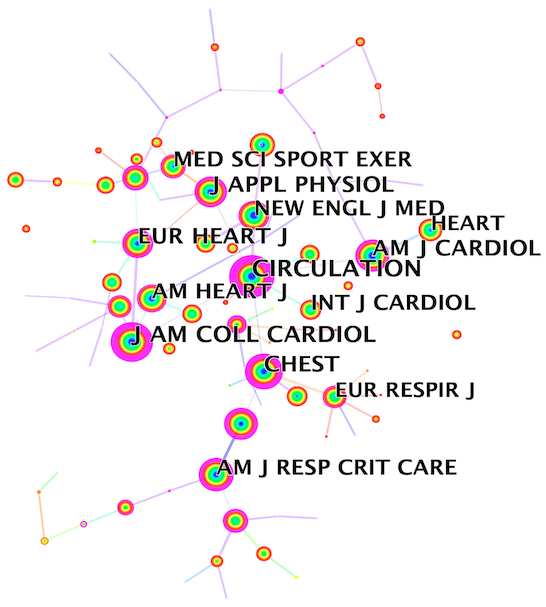

Supplement: Supplementary Figure 2 — Journal co-cited analysis. [file Image_2.JPEG]
